# Prevalence of Hearing Loss and Hearing Aid Use Among US Medicare Beneficiaries Aged 71 Years and Older

**DOI:** 10.1001/jamanetworkopen.2023.26320

**Published:** 2023-07-28

**Authors:** Nicholas S. Reed, Emmanuel E. Garcia-Morales, Clarice Myers, Alison R. Huang, Joshua R. Ehrlich, Olivia J. Killeen, Julie E. Hoover-Fong, Frank R. Lin, Michelle L. Arnold, Esther S. Oh, Jennifer A. Schrack, Jennifer A. Deal

**Affiliations:** 1 Department of Epidemiology, Johns Hopkins Bloomberg School of Public Health, Baltimore, Maryland; 2 Department of Otolaryngology-Head and Neck Surgery, Johns Hopkins School of Medicine, Baltimore, Maryland; 3 Cochlear Center for Hearing and Public Health, Johns Hopkins Bloomberg School of Public Health, Baltimore, Maryland; 4 Department of Ophthalmology and Visual Sciences, University of Michigan, Ann Arbor; 5 Institute for Social Research, University of Michigan, Ann Arbor; 6 Department of Genetic Medicine, Johns Hopkins School of Medicine, Baltimore, Maryland; 7 Department of Communication Sciences and Disorders, University of South Florida, Tampa; 8 Department of Medicine, Johns Hopkins School of Medicine, Baltimore, Maryland

## Abstract

**Importance:**

National prevalence estimates are needed to guide and benchmark initiatives to address hearing loss. However, current estimates are not based on samples that include representation of the oldest old US individuals (ie, aged ≥80 years), who are most at-risk of having hearing loss.

**Objective:**

To estimate the prevalence of hearing loss and hearing aid use by age and demographic covariates in a large, nationally representative sample of adults aged 71 years and older.

**Design, Setting, and Participants:**

In this cohort study, prevalence estimates of hearing loss by age, gender, race and ethnicity, education, and income were computed using data from the 2021 National Health Aging and Trends Study. Survey weights were applied to produce nationally representative estimates to the US older population. Data were collected from June to November 2021 and were analyzed from November to December 2022.

**Main Outcomes and Measures:**

Criterion-standard audiometric measures of hearing loss and self-reported hearing aid use.

**Results:**

In this nationally representative sample of 2803 participants (weighted estimate, 33.1 million individuals) aged 71 years or older, 38.3% (95% CI, 35.5%-41.1%) were aged 71 to 74 years, 36.0% (95% CI, 33.1%-38.8%) were aged 75 to 79 years, 13.8% (95% CI, 12.6%-14.9%) were aged 80 to 84 years, 7.9% (95% CI, 7.2%-8.6%) were aged 85 to 89 years, and 4.0% (95% CI, 3.5%-4.6%) were aged 90 years or older; 53.5% (95% CI, 50.9%-56.1%) were female and 46.5% (95% CI, 43.9%-49.1%) were male; and 7.5% (95% CI, 6.2%-8.7%) were Black, 6.5% (95% CI, 4.4%-8.7%) were Hispanic, and 82.7% (95% CI, 79.7%-85.6%) were White. An estimated 65.3% of adults 71 years and older (weighted estimate, 21.5 million individuals) had at least some degree of hearing loss (mild, 37.0% [95% CI, 34.7%-39.4%]; moderate, 24.1% [95% CI, 21.9%-26.4%]; and severe, 4.2% [95% CI, 3.3%-5.3%]). The prevalence was higher among White, male, lower-income, and lower education attainment subpopulations and increased with age, such that 96.2% (95% CI, 93.9%-98.6%) of adults aged 90 years and older had hearing loss. Among those with hearing loss, only 29.2% (weighted estimate, 6.4 million individuals) used hearing aids, with lower estimates among Black and Hispanic individuals and low-income individuals.

**Conclusions and Relevance:**

These findings suggest that bilateral hearing loss is nearly ubiquitous among older US individuals, prevalence and severity increase with age, and hearing aid use is low. Deeper consideration of discrete severity measures of hearing loss in this population, rather than binary hearing loss terminology, is warranted.

## Key Points

**Question:**

What is the national prevalence of hearing loss among older US adults?

**Findings:**

In this cohort study of a nationally representative sample of 2803 older adults, 65.3% (representing 21.5 million individuals) of those aged 71 years or older had hearing loss, and by age 90 years, 96.2% of adults had hearing loss. However, only 29.2% of those with hearing loss used hearing aids.

**Meaning:**

These findings suggest that hearing loss among the oldest old adults (ie, aged ≥80 years) is more pervasive than previously thought and warrants deeper consideration of discrete severity measures of hearing loss in this population, rather than binary hearing loss terminology.

## Teaser

This cohort study estimates the prevalence of hearing loss and hearing aid use by age and demographic covariates in a large, nationally representative sample of US adults aged 71 years and older.

## Introduction

Hearing loss has gained public health interest as an important factor affecting healthy aging. It is associated with cognitive decline,^1^ impaired physical function,^2^ poorer health resource utilization,^3^ and dementia.^4^ Importantly, evidence suggests that hearing treatment may mitigate these associations and prevent poor health outcomes,^4,5^ spurring federal agency actions and Congressional legislation to address hearing loss in the US.^6,7^ To this end, accurate and complete national hearing loss prevalence estimates are important for public health planning and understanding.

However, nationally representative estimates of hearing loss among older adults, particularly those older than 80 years, are limited. National estimates are from the National Health and Nutritional Examination Surveys (NHANES), nearly a decade old, and not proactively sampled to include a representative sample of the oldest old (ie, aged ≥80 years). NHANES data collection methods may also pose barriers for those with travel restrictions or who are homebound, excluding their participation.^8,9,10^ Other commonly used national estimates of hearing loss are based on self-reports^11,12^ that may lack sensitivity and specificity compared with criterion-standard hearing measures, especially for older adults, who tend to underestimate their level of hearing loss.^13^

The National Health and Aging Trends Study (NHATS)^14^ is a large, nationally representative panel study of Medicare beneficiaries aged 65 years and older with robust oversampling of the oldest old. Following a 2019 pilot,^15^ the 2021 NHATS round 11 protocol added pure-tone audiometry, the criterion standard for quantifying audiometric hearing loss, in addition to already collected self-reported hearing aid use. Using these data, we present the largest and most recent epidemiological estimates of measured hearing loss and hearing aid use among older adults in the US.

## Methods

### Study Participants

NHATS is a nationally representative longitudinal panel study of Medicare beneficiaries aged 65 years and older in the continental US (NHATS does not recruit in Alaska, Hawaii, or US territories such as Puerto Rico) begun in 2011.^14^ Although initial sampling was among community-dwelling adults, annual follow-up interviews are completed regardless of residential status (eg, institutional residence). The last sample replenishment occurred in 2015; hence, the current participants are aged 71 years and older. NHATS oversamples by age and race to provide statistical power for analyses of commonly underrepresented populations and uses in-home visits to overcome travel restrictions. NHATS data are publicly available. Data collection protocols were approved by the Johns Hopkins University institutional review board, and all participants provided informed consent. This cohort study follows the Strengthening the Reporting of Observational Studies in Epidemiology (STROBE) reporting guidelines for observational studies.

In round 11 of NHATS, 2803 of 3817 surveyed participants completed the hearing measures. Participants without hearing measures were excluded from the final estimates. For estimates stratified by demographic and socioeconomic variables, an additional 30 participants had data missing for race or ethnicity, 26 had data missing for education level, and 2 had income data missing.

### Hearing Measurement

Pure-tone air conduction audiometry was conducted in participants’ homes using an iPad (Apple)–based portable audiometer (SHOEBOX Ltd) with sound-attenuating Sennheiser DD450 headphones.^16,17^ Trained technicians used a software-assisted algorithm that mimics clinical best-practice guidelines to identify the lowest volume, measured in decibels hearing level (dBHL), at which a participant could reliably respond to the presented tones. Data were collected in each ear at 0.5, 1.0, 2.0, 4.0, and 8.0 kHz. Before and during the assessment, the audiometer software continuously monitors the ambient environmental noise to ensure that noise levels do not interfere with validity of the assessment. In addition, the software monitors responses for false-positives and flags implausible or rare results.

Consistent with previous population-level definitions of hearing loss, a pure-tone average of the frequencies most important for speech understanding (0.5, 1.0, 2.0, and 4.0 kHz) was derived for the better-hearing ear (ie, hearing loss in both ears) and worse-hearing ear (ie, hearing loss in at least 1 ear). We categorized pure-tone average using the clinical cut points most used in population-level research (no hearing loss, ≤25 dBHL; mild, 26-40 dBHL; moderate, 41-60; and severe or greater, >60 dBHL).^8,9,10,18^ In sensitivity analyses, we categorized pure-tone average using the recently adopted World Health Organization categories (no hearing loss, <20 dBHL; mild, 20-34.9 dBHL; moderate, 35-49.9 dBHL; moderately severe, 50-64.9 dBHL; severe or greater, ≥65 dBHL).^18^ Hearing aid use was defined by the question, “In the last month, have you used a hearing aid or other hearing device?” with yes or no as the answer.

### Covariates

Age was categorized in 5-year bins (71-74, 75-79, 80-84, 85-89, and ≥90 years). Sociodemographic covariates included self-reported gender (female and male), race and ethnicity (Black, non-Hispanic; Hispanic; and White, non-Hispanic, hereafter referred to as *Black*, *Hispanic*, and *White*, respectively), highest education level (high school or less, some college, and college diploma or more), and income with respect to the Federal Poverty Level (FPL) for a household with at least 2 people aged 65 years or older (<100% FPL, 100%-200% FPL, and >200% FPL).

### Statistical Analysis

We compared differences in demographic and socioeconomic variables between NHATS participants who did and did not complete hearing assessment using χ^2^ tests (categorical variables) and Kruskal-Wallis analysis of variance (continuous variables) to understand how well the analytic sample represents the entire study sample and inform interpretation (generalizability) of findings. Statistical significance was set at 2-sided *P* < .05. Next, among those with hearing measures, we estimated the prevalence of better-ear hearing loss (ie, hearing loss in both ears) among adults aged 71 years and older by demographic and socioeconomic variables by standardizing weighted prevalence estimates by age and gender to the population aged 71 years and older living in the continental US using data from the 2020 Census Bureau, American Community Survey, per NHATS guidelines.^14,19^ Overall prevalence estimates were calculated by all 5-year age strata. However, because of limitations in Census estimates and minimum cell size requirements, estimates that were further stratified by other demographic variables, as well as reporting of absolute number (in millions), were restricted to ages 71 to 74, 75 to 79, 80 to 84, and 85 or older years. Subsequent analyses estimated national prevalence of hearing loss by better-hearing and worse-hearing ear by age category and by hearing severity (better-ear) by age category. Finally, we calculated prevalence estimates of hearing aid use for adults aged 71 years and older with hearing loss. Population numbers were calculated from the estimated population of older adults in the continental US extrapolating the estimated prevalence of hearing loss among NHATS participants.

NHATS weights are calculated to account for differential probabilities of selection and nonresponse, and survey design, to make nationally representative parameter estimates. Analyses used round 11 survey weights. Data were collected from June to November 2021 and were analyzed from November to December 2022 using Stata statistical software version 17 (StataCorp).

## Results

Compared with 2803 participants who completed hearing testing, 1014 participants who did not complete testing were older (4.0% [3.5%-4.6%] vs 13.5% [95% CI, 10.5%-16.5%] were aged ≥90 years), more likely to be Black (7.5% [95% CI, 6.2%-8.7%] vs 9.9% [95% CI, 7.6%-12.3%]) or Hispanic (6.5% [95% CI, 4.4%-8.7%] vs 8.4% [95% CI, 5.2%-11.6%]), and more likely to have lower income levels (11.3% [95% CI, 9.5%-13.1%] vs 30.2% [95% CI, 25.0%-35.4%] had incomes <100% FPL) (Table 1). Our analytic sample included 2803 NHATS participants (2620 community dwelling and 183 institutionalized), representing 33.1 million adults aged 71 years and older. In the weighted sample, 38.3% (95% CI, 35.5%-41.1%) were aged 71 to 74 years, 36.0% (95% CI, 33.1%-38.8%) were aged 75 to 79 years, 13.8% (95% CI, 12.6%-14.9%) were aged 80 to 84 years, 7.9% (95% CI, 7.2%-8.6%) were aged 85 to 89 years, and 4.0% (95% CI, 3.5%-4.6%) were aged 90 years and older; 53.5% (95% CI, 50.9%-56.1%) were female and 46.5% (95% CI, 43.9%-49.1%) were male; and 7.5% (95% CI, 6.2%-8.7%) were Black, 6.5% (95% CI, 4.4%-8.7%) were Hispanic, and 82.7% (95% CI, 79.7%-85.6%) were White. By education, 35.0% (95% CI, 31.5%-38.5%) completed high school or less, 30.2% (95% CI, 27.4%-33.0%) completed some college, and 34.9% (95% CI, 30.8%-39.0%) completed college or advanced degrees. Finally, 11.3% (95% CI, 9.5%-13.1%) had incomes less than 100% of the FPL, 21.9% (95% CI, 19.2%-24.5%) had incomes 100% to 200% of the FPL, and 66.8% (95% CI, 63.5%-70.1%) had incomes greater than 200% of the FPL.

**Table 1.** Unweighted and Weighted Demographic Characteristics of 2021 National Health Aging Trends Study Participants With and Without Hearing Data^a^

Characteristic	Unweighted estimates, participants, No. (%) (N = 3817)			Weighted estimates, % (95% CI)		
	No hearing data	Hearing data	*P* value	No hearing data	Hearing data	*P* value
Total No.	1014	2803	NA	33.0 (29.9-36.2)	67.0 (63.8-70.1)	NA
Age, y^b^						
71-74	52 (5.1)	393 (14.0)	<.001	28.3 (21.9-34.7)	38.3 (35.5-41.1)	<.001
75-79	143 (14.1)	844 (3.1)		32.4 (27.5-37.4)	36.0 (33.1-38.8)	
80-84	137 (13.5)	710 (25.3)		15.7 (12.1-19.3)	13.8 (12.6-14.9)	
85-89	133 (13.1)	518 (18.5)		10.0 (7.6-12.4)	7.9 (7.2-8.6)	
≥90	198 (19.5)	338 (12.1)		13.5 (10.5-16.5)	4.0 (3.5-4.6)	
Gender						
Male	363 (35.8)	1204 (43.0)	<.001	42 (36.7-47.2)	46.5 (43.9-49.1)	.07
Female	651 (64.2)	1599 (57.0)		58 (52.8-63.3)	53.5 (50.9-56.1)	
Race and ethnicity^c^						
Black	241 (24.3)	529 (19.1)	<.001	9.9 (7.6-12.3)	7.5 (6.2-8.7)	.15
Hispanic	65 (6.6)	119 (4.3)		8.4 (5.2-11.6)	6.5 (4.4-8.7)	
White	657 (66.4)	2060 (74.3)		77.2 (72.9-81.6)	82.7 (79.7-85.6)	
Other^d^	27 (2.7)	65 (2.3)		4.4 (1.6-7.2)	3.3 (2.0-4.6)	
Education^e^						
High school or less	492 (51.9)	1133 (4.8)	<.001	49.8 (44.3-55.3)	35.0 (31.5-38.5)	<.001
Some college	247 (26.1)	760 (27.4)		27.5 (22.7-32.4)	30.2 (27.4-33.0)	
College or more	209 (22.0)	884 (31.8)		22.7 (17.9-27.5)	34.9 (30.8-39.0)	
Income, % of Federal Poverty Level^f^						
<100	294 (31.2)	451 (16.1)	<.001	30.2 (25.0-35.4)	11.3 (9.5-13.1)	<.001
100-200	251 (26.6)	657 (23.5)		24.2 (19.0-29.4)	21.9 (19.2-24.5)	
>200	398 (42.2)	1693 (60.4)		45.7 (39.2-52.1)	66.8 (63.5-70.1)	

Abbreviation: NA, not applicable.

^a^ Data are from the National Health Aging Trends Study. Data are weighted according to study guidelines.

^b^ Of the 351 participants for whom age data were missing at this visit, all were in the missing audiometric data group.

^c^ Fifty-four participants had missing race and ethnicity information. Of those, 24 had missing audiometric data and 30 had complete audiometric data.

^d^ Other race includes Alaska Native, American Indian, Asian, Native Hawaiian, Pacific Islander, and unlisted.

^e^ Ninety-two participants had missing education data. Of those, 66 had no missing audiometric data and 26 had complete audiometric data.

^f^ Seventy-three participants had missing income data. Of those, 71 had missing audiometric data and 2 had complete audiometric data.

In weighted analyses, 65.3% (95% CI, 62.2%-68.4%), or approximately 7 in 10, of adults aged 71 years and older had hearing loss (mild, 37.0% [95% CI, 34.7%-39.4%]; moderate, 24.1% [95% CI, 21.9%-26.4%]; and severe, 4.2% [95% CI, 3.3%-5.3%]) in the better ear (ie, hearing loss in both ears), representing an estimated 21.5 million individuals. Prevalence estimates were higher when using a worse-ear (ie, loss in at least 1 ear) definition (78.1%; 25.8 million individuals). When the newly adopted World Health Organization thresholds were applied, the prevalence of hearing loss increased such that 8 in 10 US individuals aged 71 years and older had hearing loss (eTable 1 in Supplement 1). Overall, the prevalence of hearing loss increased with age. Among those aged 80 years or older, 35.8% (95% CI, 32.8%-38.9%) had mild hearing loss, 38.8% (95% CI, 35.3%-42.3%) had moderate hearing loss, and 9.6% (95% CI, 8.1%-11.3%) had severe or greater hearing loss. In specific age strata, 53.4% (95% CI, 47.2%-59.6%; 6.0 million individuals) of participants aged 71 to 74 years had hearing loss compared with 76.7% (95% CI, 73.1%-80.3%; 6.2 million individuals) of participants aged 80 to 85 years, 91.0% (95% CI, 88.1%-93.9%) of participants aged 85 to 90 years, and 96.2% (95% CI, 93.9%-98.6%) of participants aged 90 years and older (5.5 million individuals for those aged ≥85 years). Severity of hearing loss also increased with age. Among adults aged 71 to 74 years, 46.6% (95% CI, 40.4%-52.8%) had normal hearing and 1.8% (95% CI, 0.2%-3.4%) had severe or greater hearing loss, but among those aged 90 years and older, only 3.8% (95% CI, 1.4%-6.1%) had normal hearing and 21.8% (95% CI, 16.8%-26.9%) had severe or greater hearing loss (Table 2 and Figure).

**Table 2.** Prevalence and Number Estimates of Adults Aged 71 Years or Older in the US With Hearing Loss by Age and Severity, National Health Aging Trends Study^a^

Characteristic	Prevalence, % (95% CI)					Participants with hearing loss, No. (millions) (95% CI)^b^				
	Hearing loss category					Hearing loss category				
	None	Mild	Moderate	Severe or worse	Any	None	Mild	Moderate	Severe or worse	Any
Bilateral hearing loss										
Age, y										
71-74	46.6 (40.4-52.8)	35.4 (30.1-40.8)	16.1 (11.7-20.5)	1.8 (0.2-3.4)	53.4 (47.2-59.6)	5.3 (4.6-6.0)	4.0 (3.4-4.6)	1.8 (1.3-2.3)	0.2 (0.0-0.4)	6.0 (5.3-6.7)
75-79	35.4 (31.4-39.3)	39.7 (35.9-43.4)	22.1 (18.2-26.0)	2.9 (1.3-4.4)	64.6 (60.7-68.6)	3.4 (3.0-3.8)	3.8 (3.4-4.2)	2.1 (1.7-2.5)	0.3 (0.1-0.4)	6.2 (5.8-6.5)
80-84	23.3 (19.7-26.9)	38.8 (34.9-42.8)	32.2 (27.2-37.1)	5.7 (4.0-7.4)	76.7 (73.1-80.3)	1.4 (1.2-1.6)	2.4 (2.1-2.6)	2.0 (1.7-2.3)	0.3 (0.2-0.4)	4.6 (4.4-4.9)
85-89	9.0 (6.1-11.9)	38.1 (33.2-43.0)	42.8 (38.5-47.1)	10.1 (7.2-13.0)	91.0 (88.1-93.9)	0.4 (0.3-0.6)^c^	1.9 (1.7-2.2)^c^	2.8 (2.5-3.0)^c^	0.8 (0.7-1.0)^c^	5.5 (5.4-5.6)^c^
≥90	3.8 (1.4-6.1)	21.0 (16.0-26.0)	53.4 (47.0-59.8)	21.8 (16.8-26.9)	96.2 (93.9-98.6)					
Overall	34.7 (31.6-37.8)	37.0 (34.7-39.4)	24.1 (21.9-26.4)	4.2 (3.3-5.3)	65.3 (62.2-68.4)	11.4 (10.4-12.4)	12.2 (11.4-13.0)	7.9 (7.2-8.7)	1.3 (1.0-1.7)	21.5 (20.5-22.6)
Hearing loss in ≥1 ear										
Age, y										
71-74	30.8 (25.3-36.3)	40.2 (35.4-45.0)	22.8 (18.6-27.1)	6.2 (3.4-8.9)	69.2 (63.7-74.7)	3.5 (2.8-4.1)	4.5 (4.0-5.1)	2.5 (2.1-3.0)	0.7 (0.3-1.0)	7.8 (7.2-8.4)
75-79	22.5 (19.1-26.0)	39.9 (35.2-44.6)	28.1 (23.6-32.5)	9.5 (6.7-12.3)	77.5 (74.0-80.9)	2.1 (1.8-2.4)	3.8 (3.3-4.2)	2.6 (2.2-3.1)	0.9 (0.6-1.1)	7.4 (7.1-7.7)
80-84	12.5 (9.7-15.4)	35.1 (31.0-39.2)	39.2 (34.3-44.1)	13.1 (10.3-16.0)	87.5 (84.6-90.3)	0.7 (0.5-0.9)	2.1 (1.8-2.3)	2.3 (2.0-2.6)	0.8 (0.6-0.9)	5.3 (5.1-5.5)
85-89	3.8 (2.2-5.5)	29.3 (24.2-34.5)	44.6 (40.4-48.9)	22.2 (17.8-26.7)	96.2 (94.5-97.8)	0.1 (0.0-0.2)^c^	1.3 (1.1-1.6)^c^	2.8 (2.6-3.0)^c^	1.5 (1.3-1.7)^c^	5.8 (5.7-5.8)^c^
≥90	0.5 (0.0-1.1)^d^	11.9 (7.5-16.3)	53.5 (47.1-59.8)	34.2 (28.6-39.7)	99.5 (98.9-100.0)					
Overall	21.9 (19.5-24.6)	37.4 (34.8-40.0)	29.9 (27.7-32.2)	10.7 (9.2-12.5)	78.1 (75.4-80.5)	7.2 (6.4-8.1)	12.3 (11.5-13.2)	9.8 (9.1-10.6)	3.5 (0.3-4.1)	25.8 (24.9-26.6)

^a^ Data are from the National Health Aging Trends Study, 2021 cycle (N = 2803). Data were weighted according to study guidelines. Hearing loss severity is defined according to a 4-frequency (0.5, 1.0, 2.0, and 4.0 kHz) pure-tone average threshold (no hearing loss, ≤25 decibel hearing level [dBHL]; mild, 26-40 dBHL; moderate, 41-60 dBHL; and severe or greater, >60 dBHL).

^b^ Population numbers were computed using the estimated prevalence rate and the population totals by age group in the continental US according to data from the 2020 Census Bureau, American Community Survey.

^c^ Number estimates are collapsed to participants aged 85 years and older because of limited age group estimates from the Census Bureau.

^d^ Weighted estimate is based on fewer than 10 individuals in the unweighted data.

**Figure.**
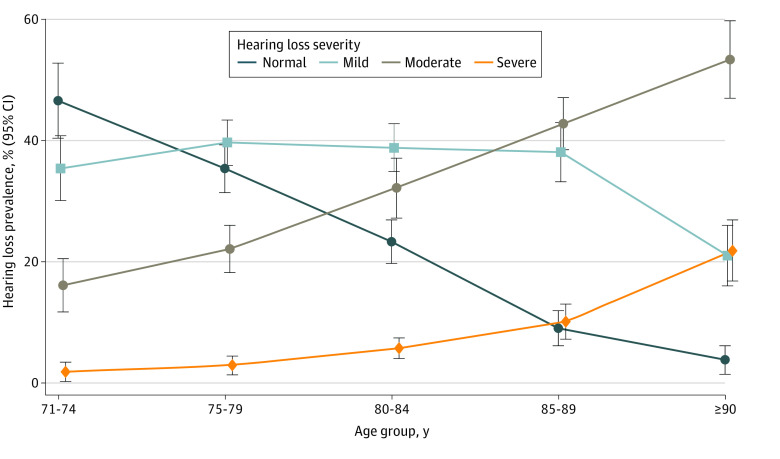
Prevalence Estimates of 2060 Adults Aged 71 Years and Older in the US With Hearing Loss by Age and Severity Data are from the 2021 National Health Aging Trends Study and are weighted according to study guidelines. Hearing loss severity is defined based on a 4-frequency (0.5, 1.0, 2.0, and 4.0 kHz) pure tone average threshold (no hearing loss, ≤25 decibel hearing level [dBHL]; mild, 26-40 dBHL; moderate, 41-60 dBHL; and severe or greater, >60 dBHL).

Male participants had a higher overall prevalence of hearing loss (69.7%; 10 million individuals) compared with female participants (61.6%; 11.4 million individuals) (Table 3). However, this difference narrowed with increasing age; the prevalence of hearing loss among participants aged 71 to 75 years was 46.5% of female participants (2.8 million individuals) vs 59.5% of male participants (3.1 million individuals). This difference was not present among participants aged 85 years and older, nearly all of whom had hearing loss (92.3% among female participants [3.5 million individuals] vs 93.8% among male participants [2.0 million individuals]). See eTable 2 in Supplement 1 for absolute number estimates by sociodemographic characteristics.

**Table 3.** Prevalence Estimates of Adults Aged 71 Years and Older in the US With Hearing Loss by Sociodemographic Characteristics and Age, National Health Aging Trends Study^a^

Age range, y	Prevalence, % (95% CI)										
	Gender		Race and ethnicity^b^			Education^c^			Income, % of Federal Poverty Level^d^		
	Female	Male	Black	Hispanic	White	High school or less	Some college	College diploma or greater	<100	100-200	>200
71-74	46.5 (39.6-53.4)	59.5 (51.4-67.5)	43.3 (32.9-53.7)	33.9 (4.9-62.9)^e^	55.5 (48.0-62.9)	62.3 (52.9-71.8)	53.1 (45.3-60.9)	47.3 (37.6-57.0)	62.6 (45.0-80.2)	66.6 (54.5-78.7)	48.3 (41.0-55.7)
75-79	60.0 (53.6-66.3)	70.2 (64.0-76.4)	51.4 (42.5-60.2)	70.4 (49.0-91.8)	66.5 (62.4-70.6)	72.3 (65.7-78.9)	60.5 (52.9-68.2)	60.1 (52.4-67.9)	61.7 (46.9-76.4)	68.6 (57.7-79.4)	64.0 (59.4-68.5)
80-84	68.5 (63.1-73.9)	87.3 (81.3-93.3)	75.2 (67.6-82.8)	86.9 (74.1-99.7)^e^	76.3 (71.9-80.7)	79.3 (73.5-85.1)	75.2 (68.2-82.1)	75.6 (69.3-81.9)	85.8 (78.3-93.2)	77.9 (70.4-85.4)	74.3 (69.5-79.1)
≥85	92.3 (89.7-94.8)	93.8 (90.9-96.7)	84.9 (78.8-91.0)	96.2 (89-100.0)^e^	93.4 (91.1-95.7)	93.6 (90.8-96.3)	96.3 (93.2-99.4)	87.9 (83.1-92.6)	93.5 (89.6-97.4)	93.7 (90.6-96.9)	92.0 (88.9-95.0)
Overall	61.6 (57.7-65.3)	69.7 (64.6-74.3)	55.8 (49.4-62.0)	59.3 (45.8-71.6)	67.0 (63.1-70.6)	74.1 (69.6-78.0)	62.3 (57.6-66.7)	59.4 (54.0-64.6)	72.7 (64.1-79.9)	72.8 (66.2-78.6)	61.6 (57.8-65.3)

^a^ Data are from the National Health Aging Trends Study, 2021 cycle (N = 2803). Data are weighted according to study guidelines.

^b^ Prevalence estimates by race and ethnicity are presented for only the 3 largest racial and ethnic groups. Individuals from all racial and ethnic groups or with missing racial and ethnic data were included in all other prevalence estimations.

^c^ Twenty-six participants had missing information regarding education level.

^d^ Two participants had missing information regarding income.

^e^ Weighted estimate is based on fewer than 10 individuals per cell either in the hearing loss or normal hearing group in the unweighted data.

White participants (67%; 18.3 million individuals) had a higher prevalence of hearing loss compared with Black (55.8%; 1.3 million individuals) and Hispanic (59.3%; 1.2 million individuals) participants. Stratified by age, the difference by race and ethnicity narrowed with increasing age in the overall sample. Among participants aged 71 to 75 years, 55.5% of White (5.2 million individuals) vs 43.3% of Black (0.3 million individuals) participants had hearing loss. However, for participants aged 85 years and older, 93.4% of White participants (4.6 million individuals) vs 84.9% of Black participants (0.3 million individuals) had hearing loss. Prevalence estimates by age were not computed for the Hispanic ethnicity category because there were fewer than 10 observations in some of the age groups. Similar trends were found by education and income levels, whereby a larger proportion of participants with lower education levels and lower income levels had hearing loss in the overall sample, but the gap narrowed with increasing age (Table 3 and eTable 2 in Supplement 1).

Among adults with hearing loss, 29.2% (6.4 million individuals) reported hearing aid use. Use increased with hearing loss severity such that 14.4% (1.7 million individuals) of those with mild, 45.3% (3.6 million individuals) of those with moderate, and 67.9% (0.9 million individuals) of those with severe hearing loss used hearing aids. A higher prevalence of hearing aid use was found among male participants (35.1%; 95% CI, 30.6%-39.9%) vs female participants (23.4%; 95% CI, 20.6%-26.4%), White (32.1%; 95% CI, 29.2%-35.1%) vs Black (8.4%; 95% CI, 5.3%-13.0%) or Hispanic (20.2%; 95% CI, 10.0%-36.7%) participants, and those with higher education and income levels (eTable 3 in Supplement 1); 35.5% (95% CI, 31.8%-3.93%) of those with hearing loss with incomes greater than 200% of the FPL used hearing aids compared with 14.6% (95% CI, 10.6%-19.7%) of those living with incomes less than 100% of the FPL. Hearing aid use also increased with age, with 31.2% (95% CI, 27.3%-35.3%) of adults aged 80 to 85 years and 36.9% (95% CI, 33.2%-40.8%) of those aged 85 years and older using hearing aids.

## Discussion

In this cohort study of a nationally representative sample of Medicare beneficiaries, nearly 7 of 10 individuals aged 71 years and older had some degree of hearing loss. Overall prevalence and severity of hearing loss increased with age such that nearly all participants had hearing loss by age 85 years. Nonetheless, less than 30% used hearing aids. To the best of our knowledge, these are the largest and most robust estimates of audiometrically measured hearing among older US individuals to date.

Compared with previous studies in nationally representative data, we report higher prevalence estimates among the oldest old individuals. Estimates from NHANES, which has limited representation over age 80 years, suggested that approximately 66%^10^ of individuals older than 70 years and 81%^8^ of individuals older than 80 years had hearing loss in the better-hearing ear. Our estimates were similar for those aged 71 years and older but higher for those aged 80 years and older. Likewise, previous estimates using the restricted 80 years and older data in NHANES suggested that 77.2% of those aged 81 to 85 years, 86.1% of those aged 85 to 90 years, and 93.8% of those aged 91 years or older have hearing loss.^8^ Our current estimates are slightly higher at 76.7% for those aged 80 to 84 years, 91.0% for those aged 85 to 89 years, and 96.2% for those aged 90 years or older. Estimates by severity were similarly higher in the current analysis (among individuals aged ≥80 years, 35.8% had mild, 38.8% had moderate, and 9.6% had severe or greater hearing loss) compared with previous work in NHANES (among individuals aged ≥80 years, 36.0% had mild, 37.9% had moderate, and 7.53% had severe or greater hearing loss).^9^ A possible explanation for the difference, especially among older age groups, is NHATS oversamples and retains underrepresented populations who are more likely to have hearing loss, including the oldest and lower income individuals. In particular, the use of home visits or facility visits for institutionalized participants in NHATS may make assessment and retention more feasible vs use of mobile testing centers in other nationally representative samples.

Our analyses shed light onto the pervasive nature of hearing loss among older adults because nearly all individuals aged 85 years and older have hearing loss. At such high proportions, severity (vs any) becomes important for characterizing hearing among older adults. Clinicians and public health officials should consider moving away from broad binary descriptions of hearing loss, especially among the oldest age groups, toward more granular categories to improve understanding and perceptions of hearing. This shift will also allow for more targeted public health planning and resource allocation, as different degrees of hearing loss may warrant different interventions and programs.^20,21^ Likewise, researchers should consider moving away from binary measurement in this age group to better understand the magnitude of the effect of differing degrees of hearing loss on poor aging outcomes.

Although the categorical definitions for hearing loss used in our study are the most common in population-level research, there is heterogeneity. In a sensitivity analysis, we applied the recently updated World Health Organization hearing loss classifications, which were recommended by an expert panel but lack details on the scientific or clinical basis for the changes. Most notably, the new classifications lowered the threshold for hearing loss from 25 dB to 20 dB.^18^ When the World Health Organization thresholds were applied, prevalence of hearing loss increased such that 8 in 10 US individuals aged 71 years and older had hearing loss (eTable 1 in Supplement 1). The nearly ubiquitous nature of hearing loss among older adults may warrant considerations for future work characterizing clinical and epidemiologic cut points specific to older adults according to what levels are important to health outcomes in this population. Importantly, the longitudinal and nationally representative nature of the publicly available NHATS hearing data and robust measures of health important to aging render it a strong data set for future work in this area.

Our study agrees with previous findings^10,22,23,24^ that prevalence of hearing loss increases with age and is highest among male, White, and lower-income individuals and those with lower educational attainment. Our findings add to the literature by demonstrating that the differences narrow as age increases. Differences in prevalence of hearing loss by demographic subgroups may be due to differences in life course noise exposure, poorer overall health among economically disadvantaged individuals, and biological factors, including the protective effects of estrogen and melanin in the cochlea.^22,23,24,25^ Although these are important considerations for public health interventions (eg, limiting noise exposure) or informing potential pharmacologic interventions to treat or prevent hearing loss (eg, melanin and estrogen), they have little implication for immediate clinical and public health interventions to address hearing loss among older adults. First, we showed that as age increased, the difference in prevalence of hearing loss among demographic subgroups narrowed to nearly no difference. Second, the negative impacts of hearing loss are similar regardless of demographic groups, and the highly prevalent nature of hearing loss warrants scoping public health approaches.

Similar to other studies, hearing aid use was relatively low, at only 29.2% of older adults with hearing loss. Previous estimates using NHANES reported that 17.6% of all adults aged 80 years and older use hearing aids (approximately 22% of those ≥80 years with hearing loss when extrapolating from published tables).^8^ Our work limited prevalence estimates to those with hearing loss (eg, at risk for hearing aid use) and found larger proportions of hearing aid use in the strata of those aged 80 years and older: 31.2% for adults aged 80 to 85 years and 36.9% for those aged 85 years and older. Although NHATS’s unique sampling strategy may contribute to differences, the observed increase in proportion of hearing aid owners in this population may also reflect the increasing trend in overall uptake in hearing aids.^26^ Our findings that race and ethnicity, education, and income levels were associated with lower prevalence of hearing aid use are consistent with previous studies in representative and population-based samples.^26,27,28^ As use of other technologies (eg, cochlear implants and over-the-counter hearing aids) increase, future work could consider additional questions to further characterize the use of hearing care in the US.

The presented data have implications for recent hearing-focused policy and initiatives. The 2017 Over-the-Counter Hearing Aid Act became official following the US Food and Drug Administration final regulations that created a regulated over-the-counter category of hearing aids for mild-to-moderate hearing loss.^7^ In addition, addressing hearing loss to reduce dementia has been added to the National Plan to Address Alzheimer Disease,^6^ and Medicare^20^ coverage of hearing aids was included in the proposed 2021 Build Back Better bill in the US Congress. Accurate prevalence estimates are essential, because proportional estimates off by just a few percentage points could reflect large differences (on the order of millions of individuals) when extrapolating to absolute numbers for the US population. In the future, larger samples will increase the precision of hearing estimates among the oldest old, but the current data offer a first step in the direction of more accurate estimates.

### Limitations

Although there are strengths of NHATS data, including the large sample size and oversampling of older individuals, the analysis has limitations. Groups at the highest risk for hearing loss are more likely to be missing hearing assessment data, which could suggest that the data presented in this analysis underestimate hearing loss prevalence. In addition, current estimates are cross-sectional; nationally representative data on the incidence and timing of hearing loss across the life course are lacking. Future work should provide temporal incidence measures. Moreover, even though NHATS is designed to be nationally representative, there are some limitations in the generalizability. The presented data are only for adults aged 71 years and older because the last replenishment occurred in 2015 (data for individuals aged ≥65 years will be available in the next replenishment scheduled for 2022-2023). Only the continental US states are sampled. Although the NHATS data oversample Black individuals, other racial and ethnic groups are not oversampled because of the relatively low population totals among older individuals. Therefore, we were not able to calculate prevalence estimates beyond White, Black, and Hispanic individuals. However, additional iterations of NHATS will allow for analyses of other subpopulations and racial and ethnic groups. Notably, in keeping with the changing demographics of the US, NHATS plans to oversample Hispanic individuals in the next replenishment. Hearing aid data are in the context of the use of hearing aids in the past month and do not offer details into total length of use or allow for estimates of overall ownership of hearing aids regardless of use, which may be helpful for policy analyses moving forward. The use of income among older adults is limited because it may not fully encapsulate socioeconomic status among older individuals; many are retired and have various sources of wealth. Although efforts are made to include all sources of income among participants, cautious interpretation is warranted. Furthermore, although NHATS offers several first-in-kind estimates in specific demographic groups (eg, adults aged >90 years), there is still imprecision in the estimates owing to the width of the intervals, and caution should be used when applying the estimates.

## Conclusions

This study found that approximately two-thirds of adults older than 71 years and nearly all adults older than 85 years have hearing loss, but relatively few use hearing aids. These robust estimates from a nationally representative study that oversamples older adults are higher than previous estimates and provide updated metrics for resource planning related to ongoing and future hearing policy initiatives.

## Notes

Supplement 1. **eTable 1.** Prevalence Estimates of Adults ≥71 Years in the United States With Hearing Loss According to Updated World Health Organization Hearing Severity Categories^a^ by Age: National Health Aging Trends Study^b^ **eTable 2.** Number Estimates of Adults ≥71 Years in the United States With Hearing Loss by Sex, Race/Ethnicity, and Age: National Health Aging Trends Study^a^ **eTable 3.** Prevalence and Number Estimates of Hearing Aid Use Among Adults ≥71 Years With Hearing Loss in the United States by Select Sociodemographic Characteristics: National Health Aging Trends Study^a^

Supplement 2.
Data Sharing Statement
