# Supplementary material for: Prevalence of Hearing Loss and Hearing Aid Use Among US Medicare Beneficiaries Aged 71 Years and Older
Source: JAMA Netw Open. 2023 Jul 28;6(7):e2326320. doi: 10.1001/jamanetworkopen.2023.26320 (PMC10383002; doi:10.1001/jamanetworkopen.2023.26320)
Supplement: Supplement 1. — eTable 1. Prevalence Estimates of Adults ≥71 Years in the United States With Hearing Loss According to Updated World Health Organization Hearing Severity Categoriesa by Age: National Health Aging Trends Studyb eTable 2. Number Estimates of Adults ≥71 Years in the United States With Hearing Loss by Sex, Race/Ethnicity, and Age: National Health Aging Trends Studya eTable 3. Prevalence and Number Estimates of Hearing Aid Use Among Adults ≥71 Years With Hearing Loss in the United States by Select Sociodemographic Characteristics: National Health Aging Trends Studya [file jamanetwopen-e2326320-s001.pdf]

Supplemental Online Content

Reed NS, Garcia-Morales EE, Myers C, et al. Prevalence of hearing loss and hearing aid use among US Medicare beneficiaries aged 71 years and older. *JAMA Netw Open*. 2023;6(7):e2326320. doi:10.1001/jamanetworkopen.2023.26320

eTable 1. Prevalence Estimates of Adults ≥71 Years in the United States With Hearing Loss According to Updated World Health Organization Hearing Severity Categories[a] by Age: National Health Aging Trends Study[b]

eTable 2. Number Estimates of Adults ≥71 Years in the United States With Hearing Loss by Sex, Race/Ethnicity, and Age: National Health Aging Trends Study[a]

eTable 3. Prevalence and Number Estimates of Hearing Aid Use Among Adults ≥71 Years With Hearing Loss in the United States by Select Sociodemographic Characteristics: National Health Aging Trends Study[a]

This supplemental material has been provided by the authors to give readers additional information about their work.

© 2023 Reed NS et al. *JAMA Network Open*.

eTable 1. Prevalence Estimates of Adults ≥71 Years in the United States With Hearing Loss According to Updated World Health Organization Hearing Severity Categories[a] by Age: National Health Aging Trends Study[b]

	Prevalence, % (95% CI)				
	Hearing Loss Category				
Age, y	Mild	Moderate	Moderately-Severe	≥Severe	Any HL
71-74y	48.9 (43.9, 53.8)	21.6 (16.9, 26.3)	5.7 (2.8, 8.5)	0.8 (-0.1, 1.7) [c]	76.9 (73.1, 80.7)
75-79y	43.7 (39.2, 48.1)	28.4 (24.6, 32.3)	8.7 (6.8, 10.6)	2.1 (0.7, 3.4)	82.9 (79.5, 86.2)
80-84y	36.2 (32.0, 40.4)	35.2 (30.5, 39.9)	14.6 (11.1, 18.1)	4.0 (2.2, 5.7)	89.9 (87.1, 92.8)
85-89y	29.7 (24.5, 35.0)	39.3 (34.5, 44.1)	20.1 (16.6, 23.7)	7.8 (5.4, 10.1)	96.8 (95.4, 98.3)
≥90y	12.6 (8.5, 16.7)	36.5 (30.6, 42.3)	35.5 (29.3, 41.7)	15.1 (10.8, 19.3)	99.7 (99.1, 100)
Overall	42.3 (39.2, 45.4)	27.9 (25.4, 30.6)	10.3 (8.9, 12.0)	2.8 (2.2, 3.5)	83.3 (81.5, 85.0)

Note. CI=confidence interval; y=years; HL=Hearing loss.

Source. National Health Aging Trends Study. Data weighted according to study guidelines.

[a] Hearing loss severity is defined based on a four-frequency (0.5/1/2/4 kHz) pure tone average threshold for the better-hearing ear (no hearing loss : <20 dB HL, mild: 20-34.9 dB HL, moderate 35-49.9 dB HL, moderately severe 50-64.9 dB HL, severe or greater ≥65 dB HL)
[b] NHATS 2021 cycle N=2803
[c] Weighted estimate based on less than 10 individuals per cell either in the hearing loss or normal hearing group in the unweighted data.

© 2023 Reed NS et al. *JAMA Network Open*.

eTable 2. Number Estimates of Adults ≥71 Years in the United States With Hearing Loss by Sex, Race/Ethnicity, and Age: National Health Aging Trends Study[a]

	Number with hearing loss (millions) (95% CI)[b]				
	Sex		Race/Ethnicity[c]		
Age, y	Female	Male	White	Black	Hispanic
71-75y	2.8 (2.4, 3.2)	3.1 (2.7, 3.5)	5.2 (4.5, 5.9)	0.3 (0.2, 0.4)	0.2 (0.0, 0.5)[d]
75-80y	3.1 (2.8, 3.4)	3.0 (2.7, 3.3)	5.2 (4.8, 5.5)	0.3 (0.2, 0.3)	0.4 (0.3, 0.6)
80-85y	2.3 (2.2, 2.5)	2.2 (2.1, 2.4)	3.8 (3.6, 4.1)	0.4 (0.3, 0.4)	0.2 (0.1, 0.2)[d]
≥85y	3.5 (3.4, 3.6)	2.0 (1.9, 2.0)	4.6 (4.5, 4.8)	0.3 (0.3, 0.3)	0.3 (0.2, 0.3)[d]
Overall	11.4 (10.7, 12.1)	10.0 (9.3, 6.8)	18.3 (17.2, 19.2)	1.3 (1.2, 1.5)	1.2 (0.9, 1.5)

Note. CI=confidence interval; y=years. Hearing loss severity is defined based on a four-frequency (0.5/1/2/4 kHz) pure tone average threshold for the better-hearing ear (no hearing loss : ≤25 dB HL, mild: 26-40 dB HL, moderate: 41-60, and severe or greater >60 dB HL)

Source. National Health Aging Trends Study. Data weighted according to study guidelines.

[a] NHATS 2021 cycle N=2803
[b] Population numbers were computed using the estimated prevalence rate and the population totals by age group in the continental USA according to data from the 2020 Census Bureau, American Community Survey
[c] Prevalence estimates by race/ethnicity are only presented for the 3 largest racial/ethnic groups. Individuals from all racial/ethnic groups were included in all other prevalence estimations.
[d] Weighted estimate based on less than 10 individuals per cell either in the hearing loss or normal hearing group in the unweighted data.

© 2023 Reed NS et al. *JAMA Network Open*.

eTable 3. Prevalence and Number Estimates of Hearing Aid Use Among Adults ≥71 Years With Hearing Loss in the United States by Select Sociodemographic Characteristics: National Health Aging Trends Study[a]

	Prevalence, % (95% CI)	Number (millions) (95% CI)[b]
**Overall**	29.2 (26.6, 31.9)	6.4 (5.8, 7.1)
**Age, y**		
71-75y	28.2 (21.8, 35.5)	1.7 (1.2, 2.2)
75-80y	25.5 (22.1, 29.3)	1.6 (1.4, 1.9)
80-85y	31.2 (27.3, 35.3)	1.4 (1.2, 1.6)
≥85y	36.9 (33.2, 40.8)	2.0 (1.8, 2.2)
**Hearing Loss Severity**		
Mild	14.4 (11.1, 18.3)	1.7 (1.2, 2.2)
Moderate	45.3 (40.5, 50.1)	3.6 (3.1, 4.0)
≥ Severe	67.9 (58.8, 75.9)	0.9 (0.6, 1.1)
**Sex**		
Male	35.1 (30.6, 39.9)	3.6 (3.0, 4.1)
Female	23.4 (20.6, 26.4)	2.7 (2.4, 3.1)
**Race/Ethnicity**[c]		
White	32.1 (29.2, 35.1)	–[d]
Black	8.4 (5.3, 13.0)	–[d]
Hispanic	20.2 (10.0, 36.7)	–[d]
**Education**		
High school or less	23.2 (19.6, 27.3)	–[d]
Some college	27.4 (23.0, 32.4)	–[d]
College diploma or greater	37.5 (31.0, 44.5)	–[d]
**Income (Poverty Line)**		
<100%	14.6 (10.6, 19.7)	–[d]
100-200%	20.5 (16.4, 25.2)	–[d]
>200%	35.5 (31.8, 39.3)	–[d]

Note. CI=confidence interval; y=years. Hearing loss severity is defined based on a four-frequency (0.5/1/2/4 kHz) pure tone average threshold for the better-hearing ear ≥25 dB HL.

Source. National Health Aging Trends Study. Data weighted according to study guidelines.

[a]NHATS 2021 cycle N=2004 participants with hearing loss
[b]Population numbers were computed using the estimated prevalence rate and the population totals by age group in the continental USA according to data from the 2020 Census Bureau, American Community Survey
[c]Prevalence estimates by race/ethnicity are only presented for the 3 largest racial/ethnic groups. Individuals from all racial/ethnic groups were included in all other prevalence estimations.
[d]Did not estimate total number.

© 2023 Reed NS et al. *JAMA Network Open*.
