# Supplementary material for: Prevalence of Hearing Loss and Hearing Aid Use Among US Medicare Beneficiaries Aged 71 Years and Older
Source: JAMA Netw Open. 2023 Jul 28;6(7):e2326320. doi: 10.1001/jamanetworkopen.2023.26320 (PMC10383002; doi:10.1001/jamanetworkopen.2023.26320)
Supplement: Supplement 2. — Data Sharing Statement [file jamanetwopen-e2326320-s002.pdf]

Data Sharing Statement

Reed. Prevalence of Hearing Loss and Hearing Aid Use Among US Medicare Beneficiaries Aged 71 Years and Older. *JAMA Netw Open*. Published July 28, 2023.
doi:10.1001/jamanetworkopen.2023.26320

Data
Data available: No

Additional Information
Explanation for why data not available: Data is already publicly available
